# A prognostic model for overall survival in recurrent glioma patients treated with bevacizumab-containing therapy

**DOI:** 10.1007/s12672-024-00944-y

**Published:** 2024-03-22

**Authors:** Shanmu Jin, Wenlin Chen, Xiaopeng Guo, Hao Xing, Huiyu Yang, Qianshu Liu, Delin Liu, Kun Zhang, Hai Wang, Yu Xia, Siying Guo, Yaning Wang, Yixin Shi, Yilin Li, Yuekun Wang, Junlin Li, Jiaming Wu, Tingyu Liang, Tian Qu, Huanzhang Li, Tianrui Yang, Yu Wang, Wenbin Ma

**Affiliations:** 1 Department of Neurosurgery, Center for Malignant Brain Tumors, National Glioma MDT Alliance, Peking Union Medical College Hospital, Chinese Academy of Medical Sciences and Peking Union Medical College, Beijing, China; 2 4+4 Medical Doctor Program, Chinese Academy of Medical Sciences and Peking Union Medical College, Beijing, China; 3 China Anti-Cancer Association Specialty Committee of Glioma, Beijing, China; 4 Eight-Year Medical Doctor Program, Chinese Academy of Medical Sciences and Peking Union Medical College, Beijing, China

**Keywords:** Prognostic model, Prediction model, Overall survival, Recurrent glioma, Bevacizumab, WHO classification of CNS tumors

## Abstract

Predictive markers and prognostic models are useful for the individualization of cancer treatment. In this study, we sought to identify clinical and molecular factors to predict overall survival in recurrent glioma patients receiving bevacizumab-containing regimens. A cohort of 102 patients was retrospectively collected from June 2011 to January 2022 at our institution. A nomogram was generated by Cox regression and feature selection algorithms based on 19 clinicopathological and 60 molecular variables. The model's performance was internally evaluated by bootstrapping in terms of discrimination and calibration. The median overall survival from the initiation of bevacizumab administration to death or last follow-up was 11.6 months (95% CI: 9.2–13.8 months) for all 102 patients, 10.2 months (95% CI: 6.4–13.3 months) for 66 patients with grade 4 tumors, and 13.8 months (lower limit of 95% CI: 11.5 months) for 36 patients with tumors of grade lower or not available. In the final model, a lower WHO 2021 grade (Grade lower or not available vs. Grade 4, HR: 0.398, 95% CI: 0.223–0.708, p = 0.00172), having received adjuvant radiochemotherapy (Yes vs. No, HR: 0.488, 95% CI: 0.268–0.888, p = 0.0189), and wildtype EGFR (Wildtype vs. Altered, HR: 0.193, 95% CI: 0.0506–0.733, p = 0.0157; Not available vs. Altered, HR: 0.386, 95% CI: 0.184–0.810, p = 0.0118) were significantly associated with longer overall survival in multivariate Cox regression. The overall concordance index was 0.652 (95% CI: 0.566–0.714), and the areas under the time-dependent curves for 6-, 12-, and 18-month overall survival were 0.677 (95% CI: 0.516–0.816), 0.654 (95% CI: 0.470–0.823), and 0.675 (95% CI: 0.491–0.860), respectively. A prognostic model for overall survival in recurrent glioma patients treated with bevacizumab-based therapy was established and internally validated. It could serve as a reference tool for clinicians to assess the extent the patients may benefit from bevacizumab and stratify their treatment response.

**Supplementary Information:**

The online version contains supplementary material available at 10.1007/s12672-024-00944-y.

## Introduction

Gliomas are the most common primary tumors in the central nervous system (CNS). In the 5th edition of the World Health Organization (WHO) classification of CNS tumors, gliomas are divided into 4 major groups: adult-type diffuse gliomas, pediatric-type diffuse low-grade gliomas, pediatric-type diffuse high-grade gliomas, and circumscribed astrocytic gliomas [1]. Despite the development of multimodal treatments including surgery, radiotherapy, chemotherapy, targeted therapy, and immunotherapy, the recurrence rate of glioma remains high. For patients with low-grade glioma, about 60% experience recurrence within 5 years [2], while approximately 40% of grade III gliomas and 90% of grade IV gliomas progress within 2 years [3]. Unfortunately, no standard of care has been established for recurrent gliomas [4, 5].

Bevacizumab (BEV), a humanized monoclonal antibody targeting vascular endothelial growth factor (VEGF), was granted accelerated approval by the United States Food and Drug Administration (FDA) in 2009, given the high response rates and the progression-free survival (PFS) benefit in two uncontrolled phase II clinical trials [6, 7]. Since then, BEV has been increasingly used to treat recurrent gliomas [8]. The first randomized controlled phase II BELOB trial on BEV in recurrent glioblastoma (GBM) showed promising results, with 9-month overall survival (OS) reported 43% for lomustine (CCNU), 38% for BEV and 63% for the combination [9]. However, the subsequent phase III EORTC 26101 trial failed to validate the OS benefit by comparing the combination of CCNU and BEV with CCNU despite improved PFS [10]. The other trials demonstrated no survival benefit with BEV at all, either alone [11] or in combination with other agents such as irinotecan (CPT-11) [12, 13], carboplatin (CBP) [14], CCNU [15, 16], and temozolomide (TMZ) [13]. As with a recent scoping review and evidence map on BEV use in recurrent GBM, OS benefits from BEV-containing regimens still could not be verified, and BEV application in recurrent GBM was only supported by PFS benefits and side effects control [17]. Additionally, BEV did not prolong PFS or OS in patients with recurrent grade II or III glioma [18]. In a word, no prospective study of BEV in recurrent gliomas has reported an OS improvement. As a result, the primary value of BEV in managing recurrent gliomas that authoritative guidelines have recognized is the supportive care of transient symptom control on account of its steroid-sparing effect [4, 5].

Alternatively, multiple retrospective studies investigated clinical and molecular factors influencing the outcome of BEV therapy in patients with recurrent glioma outside clinical trials to identify a subpopulation of patients with improved OS and aid treatment decisions [19–24]. The common nature of these studies is that they focused on a single group of tumors (recurrent GBM [19–22, 24] or grade III glioma [23]) with one or two BEV-based regimens (BEV alone [22], BEV + CPT-11 [19, 21, 23], BEV + carmustine [BCNU] [24], or BEV ± CPT-11 [20]). Therefore, their results may not fully reflect the real-world scenario in daily clinical practice where recurrent tumors secondary to gliomas of all WHO grades may be subjected to BEV-based treatment. In contrast, we retrospectively analyzed clinical and molecular variables beyond common factors like age, Karnofsky performance status (KPS), isocitrate dehydrogenase (IDH) mutation, and O6-methylguanine-DNA methyltransferase (MGMT) promoter methylation for a prognostic model regarding OS in patients with recurrent gliomas arising from primary tumors of different grades who received various BEV-containing regimens at Peking Union Medical College Hospital (PUMCH).

## Materials and methods

### Study population

Patients with glioma who received BEV-containing therapy after recurrence at the Department of Neurosurgery at PUMCH from June 2011 to January 2022 were screened. Tumor progression was confirmed by magnetic resonance imaging (MRI) following the Response Assessment in Neuro-Oncology (RANO) criteria [25]. Eligible patients were at least 18 years of age and had undergone surgery as part of the initial treatment. Patients with spinal cord glioma or who received BEV at other centers during the disease course were excluded. Thus, a total of 102 patients were considered for subsequent analysis. This retrospective study was conducted in accordance with the Declaration of Helsinki and was approved by the Institutional Review Board of Peking Union Medical College Hospital.

### Data collection and variable processing

Demographic and clinicopathological data were collected retrospectively from medical records, including sex, age at initial diagnosis, KPS before surgery, surgery, adjuvant radiochemotherapy, time to progression, age at recurrence, surgery after recurrence, radiotherapy after recurrence, non-BEV therapy after recurrence, BEV-based therapy after recurrence, time from recurrence to BEV use, KPS before BEV use, and glucocorticoid (GC) use during BEV-based therapy. Surgery was grouped into two categories, either biopsy or resection, with patients who underwent partial or total resection pooled together. Adjuvant radiochemotherapy, surgery after recurrence, radiotherapy after recurrence, non-BEV therapy after recurrence, and GC use during BEV-based therapy were treated as binary variables, with patients who received the corresponding treatment labeled "Yes" and the others "No." BEV-based therapy after recurrence was dichotomized into two groups, single-agent BEV and combined regimen, with the most common medicines in combination being TMZ, CPT-11, nitrosoureas (CCNU and semustine [MeCCNU]), and platins (cisplatin [DDP] and CBP) (Online Resource 1).

For patients whose surgery was carried out at PUMCH, molecular testing was performed on their tumor sample. MGMT promoter methylation, IDH mutation (IDH1 R132H/IDH2 R172K), and telomerase reverse transcriptase (TERT) promoter mutation (C228T/C250T) were determined by fluorescent probe polymerase chain reaction (PCR). A next-generation sequencing (NGS) panel of 60 molecular markers, including IDH1/2, TERT, chromosome 1p/19q, cyclin-dependent kinase inhibitor 2A/B (CDKN2A/B), epidermal growth factor receptor (EGFR), phosphatase and tensin homolog (PTEN), tumor protein p53 (TP53), etc., was applied to tumor samples of sufficient quantity and quality, the complete list of which is shown in Online Resource 2. The alteration status of molecular variables was handled dichotomously, with all variants coded "altered" and the others "wildtype." In the case of all 102 patients included in the final analysis, we reassessed their primary tumor grades in accordance with the 2016 and 2021 WHO criteria respectively after thorough consultations with experienced pathologists from our institution. This re-evaluation was based on the histological descriptions provided in the original pathological reports, supplemented by any recent molecular testing results, if available.

A substantial proportion of our glioma patients came solely for second-line treatment. It is reasonable that part of their surgery- and sample-related information, e.g., pre-operative KPS, molecular alterations, and WHO 2021 grade, was irretrievable. Therefore, considering their clinical relevance, an additional category "not available" was assigned to the missing values of the abovementioned variables.

### Model development and validation

OS was defined as the time interval from the initiation of BEV administration to death due to any cause or last follow-up. A p-value less than 0.05 was considered statistically significant. The association of variables with OS was analyzed using Cox proportional hazards model. Significant variables in univariate Cox regression analysis were screened by 4 machine learning algorithms: least absolute shrinkage and selection operator (LASSO), likelihood-based gradient boosting, model-based gradient boosting, and random survival forests. The variables adopted by at least one algorithm were further selected for the final prediction model with backward elimination in multivariate Cox regression analysis. Based on the prediction model, a nomogram was built to provide visualization of the survival probabilities at 6, 12, and 18 months. Model performance was evaluated by discrimination and calibration, with internal bootstrapping of 100 resamples. Discrimination was assessed by Harrell's concordance index (c-index) and the time-dependent area under the receiver operating characteristic (ROC) curve (AUC), while calibration was evaluated using a calibration plot. All statistical analysis and graphic presentation were performed with R (version 4.3.2, R Foundation) in RStudio (version 2023.12.1 + 402, Posit Software). LASSO and model-based gradient boosting were done using the biospear package (version 1.0.2) [26], likelihood-based gradient boosting using the CoxBoost package (version 1.5) [27], and random survival forests using the randomForestSRC package (version 3.2.3) [28]. The complete R script and the anonymized patient dataset were provided in Online Resource 3.

## Results

### Patient characteristics

**Fig. 1:**
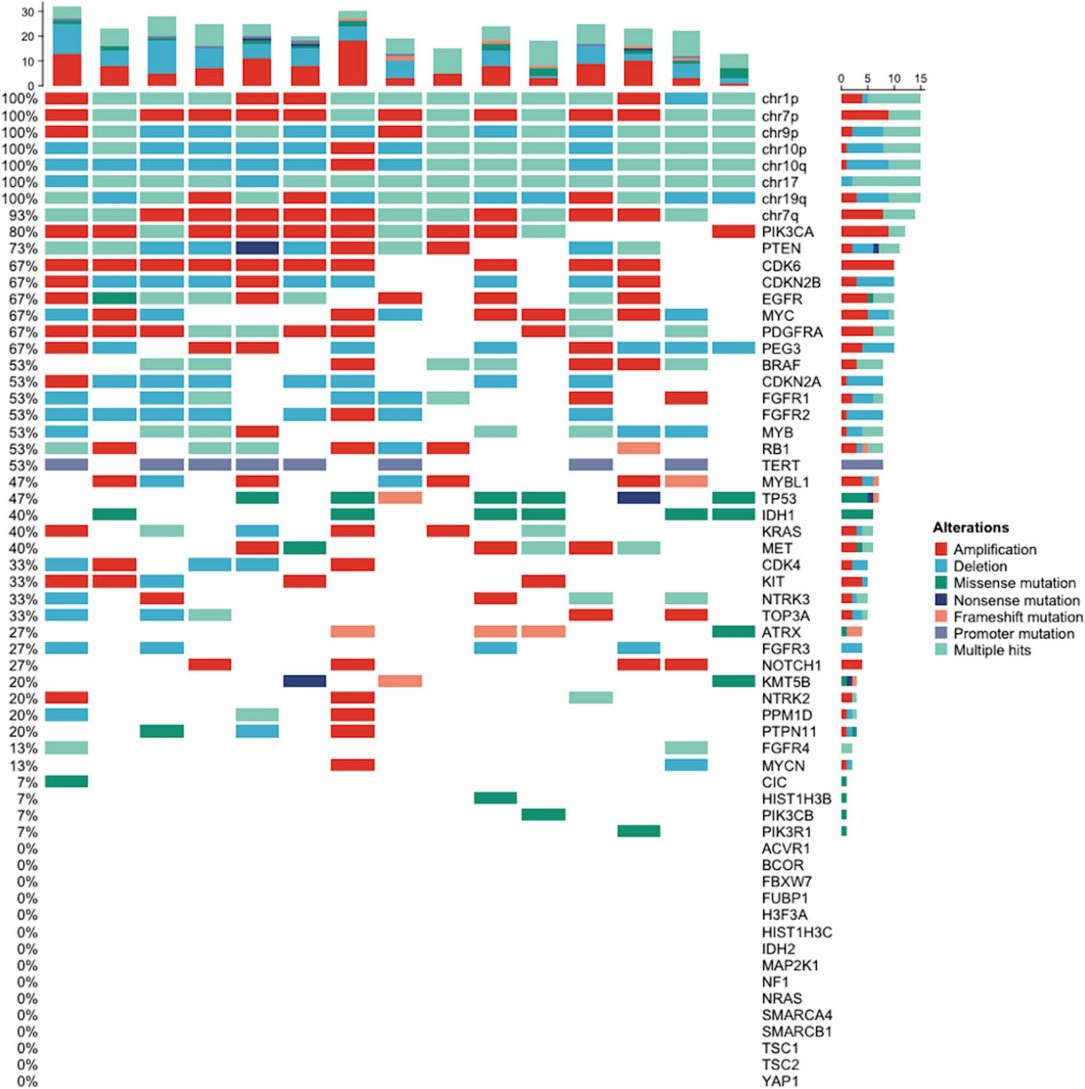
Waterfall plot of 60 molecular markers in 15 patients

The demographic and clinicopathological characteristics of the entire 102 patients are shown in Table 1. There were 58 males and 44 females. The median age at initial diagnosis was 49.5 years. Regarding 38 patients whose KPS before surgery was known, 10 were <  = 70, and the remainder were > 70. Surgery consisted of partial or total resection in 92 patients and biopsy in 10 patients. As an adjuvant treatment, 82 patients received concurrent or sequential radiochemotherapy. The median time to progression was 10.7 months for all 102 patients, 8.9 months for 66 patients with grade 4 tumors, and 18.6 months for 36 patients with tumors of grade lower or not available. The median age at recurrence was 51.0 years. Following tumor relapse, 29 patients were treated with surgery, 35 with radiotherapy, and 47 with non-BEV medication. Fifty-two patients received single-agent BEV after recurrence, while the others received a combination regimen containing BEV (Online Resource 1). The median time between tumor recurrence and the introduction of BEV was 2.6 months, and the median KPS before BEV initiation was 80. Eighty-four patients used GC during BEV-based therapy, whereas the rest did not. MGMT methylation was found in 12 patients, IDH mutation in 9, and TERT promoter mutation in 21. There were 70 and 65 patients whose MGMT methylation and IDH and TERT promoter mutation status were unavailable, respectively. WHO 2016 grade was reported in all patients, with 17 grade II tumors, 26 grade III tumors, and 59 grade IV tumors. WHO 2021 grade was specified in 72 patients, with 2 grade 2 tumors, 4 grade 3 tumors, and 66 grade 4 tumors. Glioma subtypes of the primary tumor according to the WHO 2016 and 2021 classifications are listed in Online Resource 4. The panorama of the alterations of the 60 molecular markers in 15 patients is demonstrated in Fig. 1.

**Table 1:** Demographic and clinicopathological characteristics of the study cohort

Characteristics	Categories	N = 102
Sex, No. (%)	Male	58 (56.9)
	Female	44 (43.1)
Age at initial diagnosis, years, median (range)		49.5 (24–77)
KPS before surgery, No. (%)	< = 70	10 (9.8)
	> 70	28 (27.5)
	Not available	64 (62.7)
Surgery, No. (%)	Biopsy	10 (9.8)
	Resection	92 (90.2)
Adjuvant radiochemotherapy, No. (%)	Yes	82 (80.4)
	No	20 (19.6)
Time to progression, months, median (range)		10.7 (1.6–146.1)
Age at recurrence, years, median (range)		51.0 (24–77)
Surgery after recurrence, No. (%)	Yes	29 (28.4)
	No	73 (71.6)
Radiotherapy after recurrence, No. (%)	Yes	35 (34.3)
	No	67 (65.7)
Non-BEV therapy after recurrence, No. (%)	Yes	47 (46.1)
	No	55 (53.9)
BEV-based therapy after recurrence, No. (%)	Single-agent	52 (51.0)
	Combined	50 (49.0)
Time from recurrence to BEV use, months, median (range)		2.6 (0–44.2)
KPS before BEV use, median (range)		80 (20–100)
GC use during BEV-based therapy, No. (%)	Yes	84 (82.4)
	No	18 (17.6)
MGMT promoter methylation, No. (%)	Yes	12 (11.8)
	No	20 (19.6)
	Not available	70 (68.6)
IDH mutation, No. (%)	Yes	9 (8.8)
	No	28 (27.5)
	Not available	65 (63.7)
TERT promoter mutation, No. (%)	Yes	21 (20.6)
	No	16 (15.7)
	Not available	65 (63.7)
WHO 2016 grade, No. (%)	II	17 (16.7)
	III	26 (25.5)
	IV	59 (57.8)
WHO 2021 grade, No. (%)	2	2 (2.0)
	3	4 (3.9)
	4	66 (64.7)
	Not available	30 (29.4)

### Survival and prognostic factors

An outcome event occurred in 69 patients. The median OS was 11.6 months (95% CI: 9.2–13.8 months) for all 102 patients, 10.2 months (95% CI: 6.4–13.3 months) for 66 patients with grade 4 tumors, and 13.8 months (lower limit of 95% CI: 11.5 months) for 36 patients with tumors of grade lower or not available. All the potential factors that could be associated with OS, including the 19 characteristics in Table 1 and the 60 molecular markers in Online Resource 2, were incorporated into univariate Cox regression. WHO 2016 grade, WHO 2021 grade, adjuvant radiochemotherapy, time to progression, IDH mutation, TERT promoter mutation, cyclin-dependent kinase 6 (CDK6), CDKN2B, EGFR, fibroblast growth factor receptor 3 (FGFR3), and histone cluster 1 H3 family member B (HIST1H3B) were significantly associated with OS in the univariate analysis (Table 2). Given that the WHO 2016 criteria have been supplanted by the WHO 2021 criteria in current clinical practice, WHO 2016 grade was excluded from subsequent analyses. Due to the considerable correlation between the other 10 variables (Online Resource 5), feature selection was employed. The variables were fed separately into LASSO, likelihood-based gradient boosting, model-based gradient boosting, and random survival forests. WHO 2021 grade, adjuvant radiochemotherapy, time to progression, IDH mutation, and EGFR were reserved by all 4 methods, while each of the other 5 factors was discarded by at least one algorithm (Online Resource 6). The remaining 5 variables were involved in multivariate Cox regression, and 3 of them, namely WHO 2021 grade, adjuvant radiochemotherapy, and EGFR, were significantly associated with OS after backward elimination (Table 2).

**Table 2:** Factors significantly associated with OS in the study cohort

Factors	Categories	Univariate Cox regression		Multivariate Cox regression	
		Hazard ratio (95% CI)	P-value	Hazard ratio (95% CI)	P-value
WHO 2016 grade		1.40 (1.01–1.95)	0.0436		
WHO 2021 grade	Grade 4	1		1	
	Grade lower or not available	0.492 (0.287–0.844)	0.0100	0.398 (0.223–0.708)	0.00172
Adjuvant radiochemotherapy	Yes	0.528 (0.306–0.910)	0.0215	0.488 (0.268–0.888)	0.0189
	No	1		1	
Time to progression		0.987 (0.974–0.999)	0.0341		
IDH mutation	Yes	0.240 (0.0827–0.695)	0.00856		
	No	1			
	Not available	0.549 (0.332–0.907)	0.0193		
TERT promoter mutation	Yes	2.68 (1.25–5.72)	0.0110		
	No	1			
	Not available	1.25 (0.652–2.41)	0.498		
CDK6	Altered	1			
	Wildtype	0.239 (0.0649–0.882)	0.0317		
	Not available	0.458 (0.231–0.906)	0.0249		
CDKN2B	Altered	1			
	Wildtype	0.239 (0.0649–0.882)	0.0317		
	Not available	0.458 (0.231–0.906)	0.0249		
EGFR	Altered	1		1	
	Wildtype	0.153 (0.0413–0.569)	0.00507	0.193 (0.0506–0.733)	0.0157
	Not available	0.350 (0.176–0.698)	0.00286	0.386 (0.184–0.810)	0.0118
FGFR3	Altered	1			
	Wildtype	0.171 (0.0505–0.579)	0.00455		
	Not available	0.183 (0.0637–0.524)	0.00156		
HIST1H3B	Altered	1			
	Wildtype	0.0243 (0.00209–0.284)	0.00302		
	Not available	0.0208 (0.00189–0.230)	0.00159		

### Prediction model

**Fig. 2:**
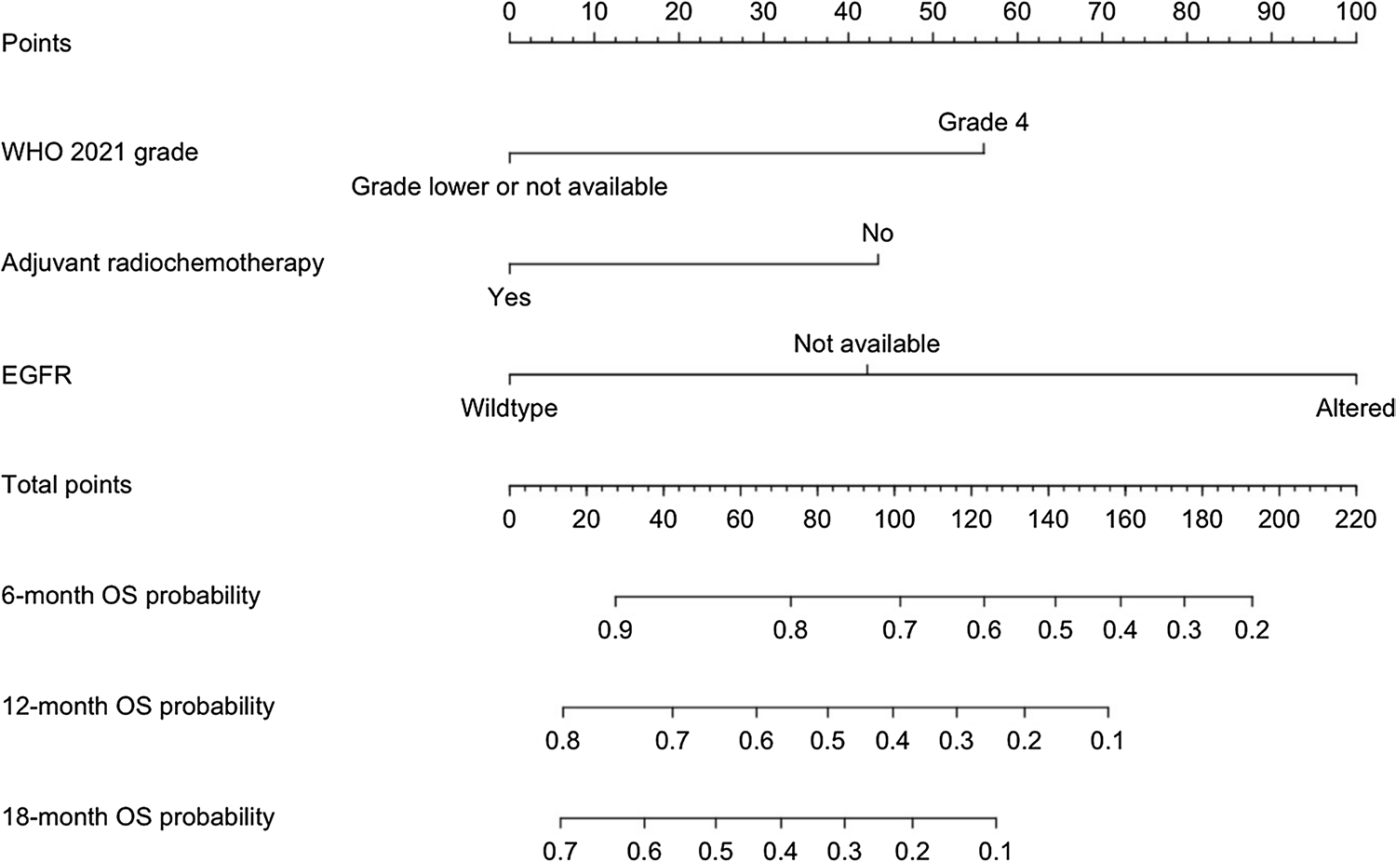
Nomogram predicting survival probability at 6, 12, and 18 months after BEV administration

**Fig. 3:**
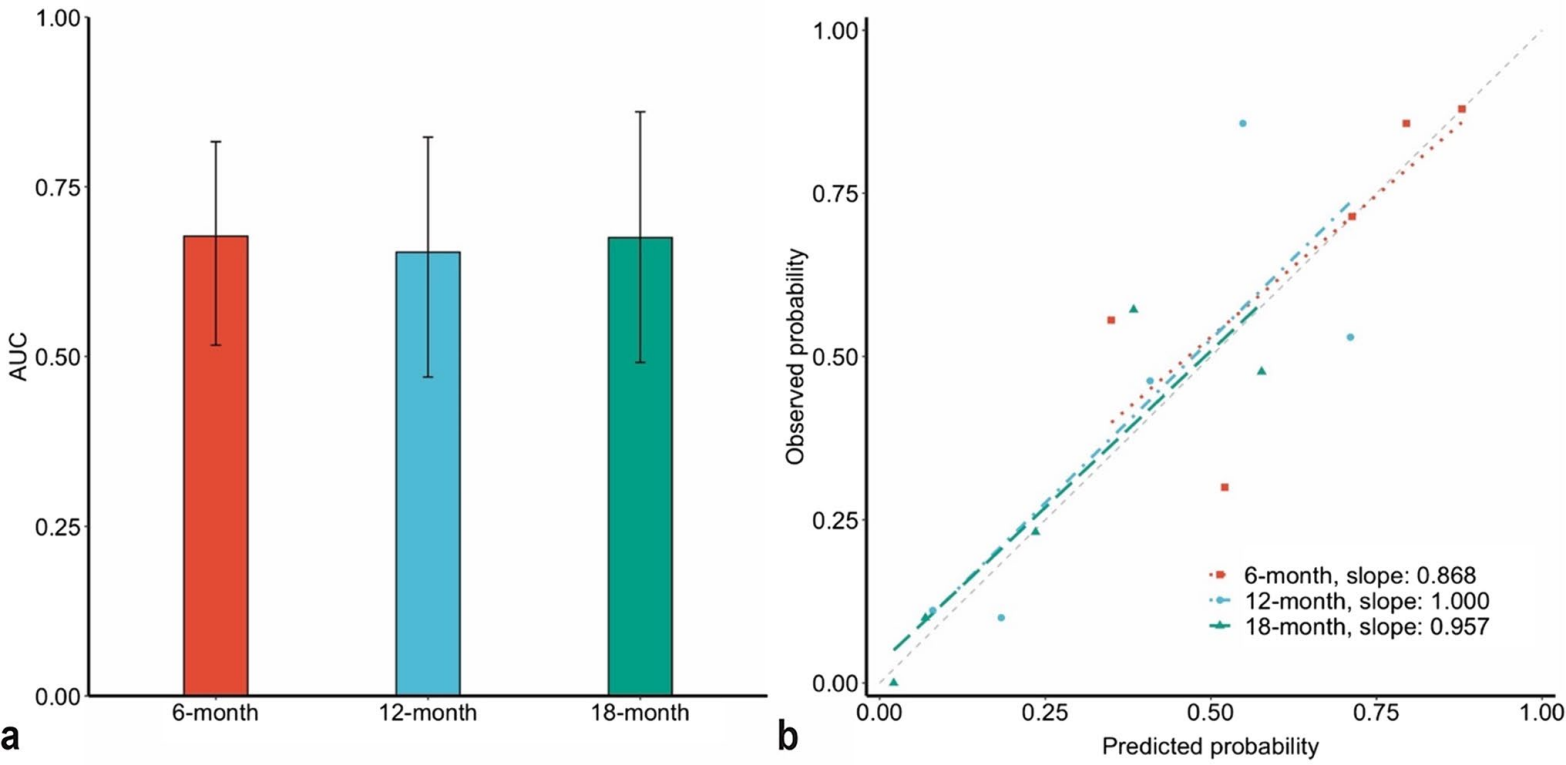
Discrimination (**a**) and calibration (**b**) of the prediction model

The nomogram was established based on the results of multivariate Cox analysis where WHO 2021 grade, adjuvant radiochemotherapy, and EGFR were utilized in the model construction (Fig. 2). It predicts the probabilities of achieving 6-, 12-, and 18-month OS in patients with recurrent glioma after the administration of BEV. The model was internally validated by bootstrapping, with an overall c-index of 0.652 (95% CI: 0.566–0.714). The time-dependent AUCs at 6, 12, and 18 months were 0.677 (95% CI: 0.516–0.816), 0.654 (95% CI: 0.470–0.823), and 0.675 (95% CI: 0.491–0.860), respectively (Fig. 3a), indicating a more consistent prediction of shorter-term OS since the lower bound of the 95% CI of the AUC at 6 months was over 0.5. The calibration plot displayed an agreement between model predictions and actual observations for 6-, 12-, and 18-month OS, with slopes around 1 and intercepts around 0 (Fig. 3b).

## Discussion

We have developed a prediction model for estimating the probability of 6-, 12-, and 18-month OS in recurrent glioma patients treated with BEV, which combines the information on adjuvant therapy, WHO 2021 grade, and EGFR alteration status. The model relies simply on treatment history and tumor pathology, so it would be convenient to use in routine clinical practice. To our knowledge, this model is the first to cover gliomas from grade 2 to 4 and patients receiving diverse BEV-containing regimens.

According to our model, recurrent glioma patients with lower grade tumors are likely to live longer than those with higher grade tumors after BEV use. This is consistent with the results of previous high-quality clinical trials comparing BEV-based therapy with non-BEV treatment [10, 11, 16, 18, 29], where recurrent GBM patients receiving BEV had a median OS of 6.4–10.0 months [10, 11, 16, 29] while the median OS in patients with recurrent grade II and III glioma using BEV was 13.8 months [18]. Nevertheless, it should not be interpreted as BEV being "more effective" in lower-grade recurrent gliomas, for these tumors have much lower microvascular proliferation and much smaller growth momentum compared to their higher-grade counterparts [30, 31].

In 2005, the seminal results of the EORTC-NCIC trial demonstrated that the addition of oral TMZ to radiotherapy increased the median OS of GBM patients to 14.6 months [32, 33]. Since then, post-operative radiochemotherapy has been established as the standard of care for GBM patients [4, 5]. Recent longitudinal data also supported the survival benefits of this treatment protocol outside clinical trials [34, 35]. Even in patients with irresectable GBM, more intense adjuvant therapy was significantly associated with longer OS, as the radiochemotherapy group had the most prolonged OS, those with monotherapy of either radiation or chemotherapeutics followed, and the shortest OS was in the group without any adjuvant treatment [36]. As for low-grade glioma patients, chemotherapy of procarbazine, CCNU, and vincristine (PCV) or TMZ in combination with radiotherapy is recommended, despite active surveillance following surgery may be applied to low-risk individuals [4, 5]. Our study extrapolated this pattern to patients with recurrent glioma receiving BEV. Mechanistically, radiochemotherapy upregulates the expression of vascular endothelial growth factor receptor 2 (VEGFR2) in glioma [37], therefore increases tumor's dependence on VEGF pathway activation and sensitivity to BEV [38, 39]. It could be suspected that the patients who have not completed radiochemotherapy, mainly because of intolerance caused by poor general condition or personal preference of not going through further first-line treatment, would experience quicker recurrence and worse response to BEV.

EGFR is the most commonly altered receptor tyrosine kinase (RTK) in GBM [40]. In addition to amplification, missense mutations R108K, A289V, and G598V were detected in our cohort, which have been experimentally confirmed to increase EGFR gene dosage and confer tumorigenicity [41]. Oncogenic EGFR activation upregulates VEGF expression by activating at least 3 different VEGF transcription activators, signal transducer and activator of transcription 3 (STAT3), specificity protein 1 (SP1), and hypoxia-inducible factors (HIFs), via the mitogen-activated protein kinase (MAPK) and phosphoinositide 3-kinase (PI3K) signaling cascades [42], thereby contributing to BEV resistance. Several previous studies discovered no significant association between EGFR status and response to BEV in recurrent glioma patients [43–45]. In contrast, one article reported that amplified EGFR was significantly associated with a shorter time to progression on BEV, defined as the time from the start of BEV treatment until clinical or radiographic progression [46]. Thus, our finding of EGFR alteration being an independent indicator of worse OS since the use of BEV is novel.

The 2021 WHO classification of CNS tumors combines histological and molecular grading, where molecular parameters can override histological findings in assigning a grade, with examples including CDKN2A and/or CDKN2B homozygous deletion in IDH-mutant astrocytoma, as well as TERT promoter mutation, EGFR amplification, and + 7/−10 chromosome copy number changes in IDH-wildtype GBM [1]. However, WHO 2021 grading poses the challenge of the availability of genotyping assays, as molecular testing may not always be readily achievable due to various reasons. By assigning a separate category for cases with unavailable molecular data, our model can still provide prognostic estimations when molecular information is insufficient.

There are several limitations to our study. First, the relatively small number of patients from a single institution of PUMCH may cause systemic bias. Second, only 15 tumor samples underwent the NGS panel testing, which limits the significance of molecular factors and the performance of our prediction model. Specifically, this limitation reduces the statistical power to detect additional significant molecular markers, thereby restricting the predictive ability of the model. However, it underscores the robust significance of the detected marker EGFR. Third, the model was not independently validated, for no similar cohort with publicly available detailed information could be referred to despite extensive search efforts. Therefore, further research is warranted to confirm the practical value of our model.

In conclusion, we developed a prognostic model of good performance for recurrent glioma patients, which provides a handy tool in the current clinical settings for evaluating the OS after the use of BEV. It would help to indicate patients who are more likely to benefit from BEV-based therapy and formulate a sensible estimation of the potential outcome of anti-angiogenic treatment.

### Supplementary Information

Additional file1 (DOCX 15 KB)

Additional file2 (DOCX 15 KB)

Additional file3 (ZIP 13 KB)

Additional file4 (DOCX 17 KB)

Additional file5 (DOCX 183 KB)

Additional file6 (DOCX 16 KB)

## Data Availability

All data generated or analyzed during this study are included in this published article and its supplementary information files which were cited as "Online Resources" in the article.
