# Supplementary material for: A prognostic model for overall survival in recurrent glioma patients treated with bevacizumab-containing therapy
Source: Discov Oncol. 2024 Mar 22;15:85. doi: 10.1007/s12672-024-00944-y (PMC10959905; doi:10.1007/s12672-024-00944-y)
Supplement: Supplementary file 1 — Additional file1 (DOCX 15 KB) [file 12672_2024_944_MOESM1_ESM.docx]

**Online Resource 1** Medications in combination with BEV

| Medications | No. ^a^ |
| --- | --- |
| Temozolomide | 18 |
| Irinotecan | 14 |
| Lomustine | 4 |
| Semustine | 8 |
| Cisplatin | 1 |
| Carboplatin | 1 |
| Camrelizumab | 1 |
| Pembrolizumab | 2 |
| Temozolomide + Cisplatin | 1 |
| Procarbazine + Lomustine + Vincristine (PCV) | 1 |
| Ifosfamide + Carboplatin + Etoposide (ICE) | 1 |

^a^ The sum of the column (52) is larger than the number of patients who received combination regimen (50) since 2 patients received 2 medications sequentially (carboplatin → irinotecan and temozolomide → semustine) to combine with BEV
