# Supplementary material for: A prognostic model for overall survival in recurrent glioma patients treated with bevacizumab-containing therapy
Source: Discov Oncol. 2024 Mar 22;15:85. doi: 10.1007/s12672-024-00944-y (PMC10959905; doi:10.1007/s12672-024-00944-y)
Supplement: Supplementary file 2 — Additional file2 (DOCX 15 KB) [file 12672_2024_944_MOESM2_ESM.docx]

**Online Resource 2** The complete list of molecular markers

| 60 molecular markers | | | | | | | | | |
| --- | --- | --- | --- | --- | --- | --- | --- | --- | --- |
| ACVR1 | ATRX | BCOR | BRAF | CDK4 | CDK6 | CDKN2A | CDKN2B | CIC | EGFR |
| FBXW7 | FGFR1 | FGFR2 | FGFR3 | FGFR4 | FUBP1 | H3F3A | HIST1H3B | HIST1H3C | IDH1 |
| IDH2 | KIT | KMT5B | KRAS | MAP2K1 | MET | MYB | MYBL1 | MYC | MYCN |
| NF1 | NOTCH1 | NRAS | NTRK2 | NTRK3 | PDGFRA | PEG3 | PIK3CA | PIK3CB | PIK3R1 |
| PPM1D | PTEN | PTPN11 | RB1 | SMARCA4 | SMARCB1 | TERT | TOP3A | TP53 | TSC1 |
| TSC2 | YAP1 | chr1p | chr7p | chr7q | chr9p | chr10p | chr10q | chr17 | chr19q |
