# Supplementary material for: A prognostic model for overall survival in recurrent glioma patients treated with bevacizumab-containing therapy
Source: Discov Oncol. 2024 Mar 22;15:85. doi: 10.1007/s12672-024-00944-y (PMC10959905; doi:10.1007/s12672-024-00944-y)
Supplement: Supplementary file 4 — Additional file4 (DOCX 17 KB) [file 12672_2024_944_MOESM4_ESM.docx]

**Online Resource 4** Glioma subtypes of the primary tumor according to the WHO 2016 and 2021 classifications

| Glioma subtypes | Grade | No. |
| --- | --- | --- |
| WHO 2016 classification |  |  |
| Diffuse astrocytoma, IDH-mutant | II | 4 |
| Diffuse astrocytoma, IDH-wildtype | II | 1 |
| Diffuse astrocytoma, NOS ^a^ | II | 5 |
| Anaplastic astrocytoma, IDH-mutant | III | 2 |
| Anaplastic astrocytoma, IDH-wildtype | III | 5 |
| Anaplastic astrocytoma, NOS | III | 8 |
| Oligodendroglioma, IDH-mutant and 1p/19q co-deleted | II | 1 |
| Oligodendroglioma, NOS | II | 2 |
| Anaplastic oligodendroglioma, NOS | III | 3 |
| Oligoastrocytoma, NOS | II | 2 |
| Anaplastic oligoastrocytoma, NOS | III | 4 |
| Glioblastoma, IDH-mutant | IV | 1 |
| Glioblastoma, IDH-wildtype | IV | 14 |
| Glioblastoma, NOS | IV | 43 |
| Diffuse midline glioma, H3 K27M-mutant | IV | 1 |
| Pleomorphic xanthoastrocytoma | II | 1 |
| Anaplastic pleomorphic xanthoastrocytoma | III | 3 |
| Diffuse glioma | II | 1 |
|  | III | 1 |
| WHO 2021 classification |  |  |
| Astrocytoma, IDH-mutant | 2 | 1 |
|  | 3 | 1 |
|  | 4 | 3 |
|  | Not available | 2 |
| Oligodendroglioma, IDH-mutant, and 1p/19q-codeleted | 2 | 1 |
| Glioblastoma, IDH-wildtype | 4 | 19 |
| Pleomorphic xanthoastrocytoma | 3 | 2 |
| Diffuse glioma, IDH-wildtype, NOS | 3 | 1 |
| NOS | 4 | 44 |
|  | Not available | 28 |

^a^ NOS: not otherwise specified
