# Supplementary material for: A prognostic model for overall survival in recurrent glioma patients treated with bevacizumab-containing therapy
Source: Discov Oncol. 2024 Mar 22;15:85. doi: 10.1007/s12672-024-00944-y (PMC10959905; doi:10.1007/s12672-024-00944-y)

**Online Resource 5** Correlation heatmap of the significant variables in univariate Cox regression


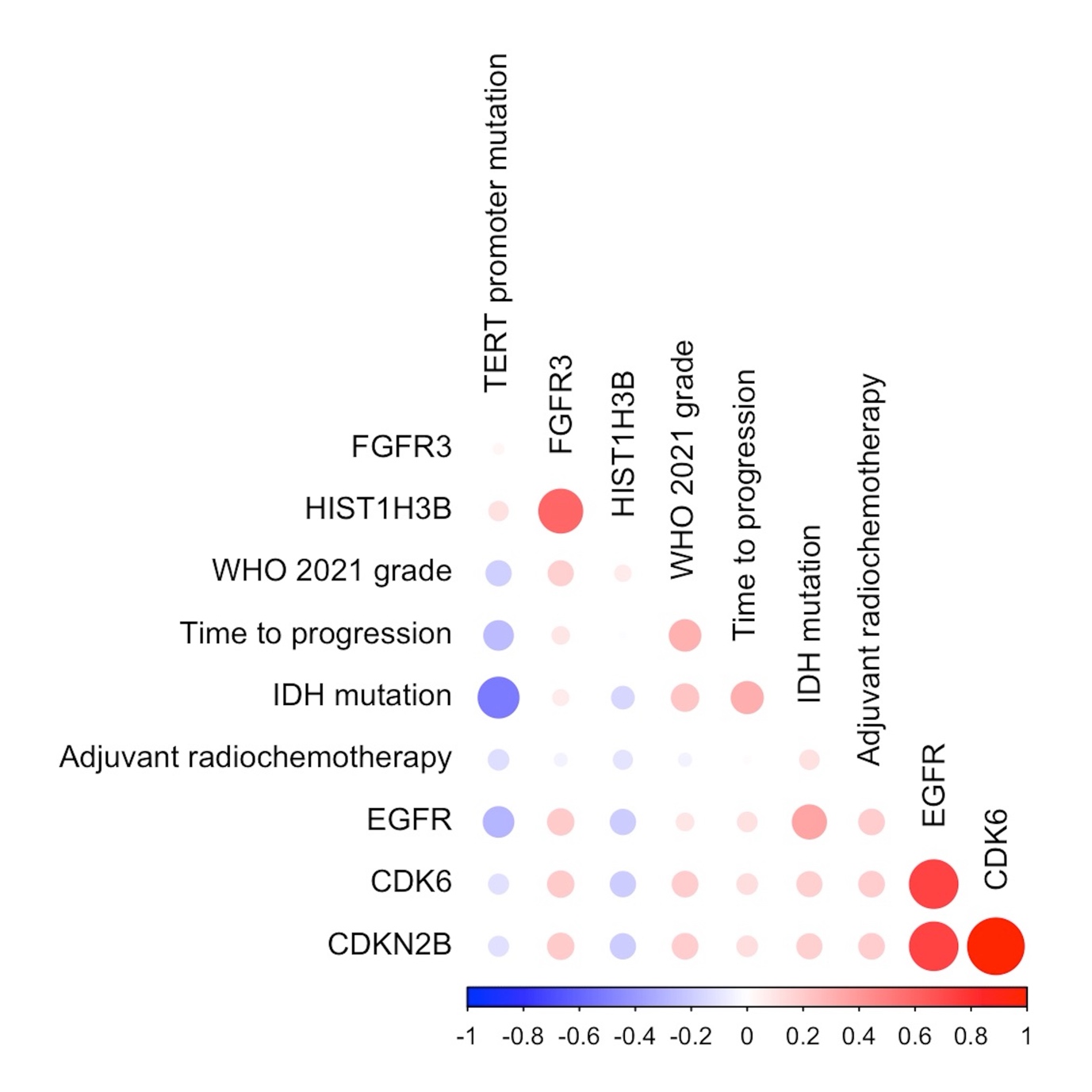

Supplement: Supplementary file 5 — Additional file5 (DOCX 183 KB) [file 12672_2024_944_MOESM5_ESM.docx]
