# Supplementary material for: A prognostic model for overall survival in recurrent glioma patients treated with bevacizumab-containing therapy
Source: Discov Oncol. 2024 Mar 22;15:85. doi: 10.1007/s12672-024-00944-y (PMC10959905; doi:10.1007/s12672-024-00944-y)
Supplement: Supplementary file 6 — Additional file6 (DOCX 16 KB) [file 12672_2024_944_MOESM6_ESM.docx]

**Online Resource 6** Feature selection using machine learning algorithms

|  | LASSO | Likelihood-based gradient boosting | Model-based gradient boosting | Random survival forests | Consensus |
| --- | --- | --- | --- | --- | --- |
| WHO 2021 grade | √ | √ | √ | √ | √ |
| Adjuvant radiochemotherapy | √ | √ | √ | √ | √ |
| Time to progression | √ | √ | √ | √ | √ |
| IDH mutation | √ | √ | √ | √ | √ |
| TERT promoter mutation | √ | √ |  | √ |  |
| CDK6 |  |  |  |  |  |
| CDKN2B |  |  |  |  |  |
| EGFR | √ | √ | √ | √ | √ |
| FGFR3 |  | √ |  | √ |  |
| HIST1H3B |  |  |  | √ |  |
